# Identify optimal HAP series scores for unresectable HCC patients undergoing TACE plus sorafenib: A Chinese multicenter observational study

**DOI:** 10.3389/fonc.2022.983554

**Published:** 2023-01-27

**Authors:** Yejing Zhu, Enxin Wang, Shoujie Zhao, Dandan Han, Yan Zhao, Hui Chen, Jun Zhu, Tenghui Han, Yang Bai, Yanju Lou, Yongchao Zhang, Man Yang, Luo Zuo, Jiahao Fan, Xing Chen, Jia Jia, Wenbin Wu, Weirong Ren, Tingting Bai, Shouzheng Ma, Fenghua Xu, Yuxin Tang, Ying Han, Junlong Zhao, Xingshun Qi, Jing Li, Xilin Du, Dongfeng Chen, Lei Liu

**Affiliations:** ^1^ Department of General Surgery, Tangdu Hospital, Air Force Medical University (Fourth Military Medical University), Xi'an, China; ^2^ Department of Digestive Diseases, Air Force Hospital of Western Theater Command, Chengdu, China; ^3^ Department of General Surgery, The Air Force Hospital of Southern Theater Command, Guangzhou, China; ^4^ Department of Digestive Diseases, The First Affiliated Hospital of Xi'an Jiao Tong University, Xi’an, China; ^5^ Xijing Hospital of Digestive Diseases, Air Force Medical University (Fourth Military Medical University), Xi’an, China; ^6^ Department of Internal Medicine, Air Force Medical University (Fourth Military Medical University), Xi’an, China; ^7^ Department of Neurosurgery, General Hospital of Northern Theater Command, Shenyang, China; ^8^ Department of Orthopedic Surgery, Air Force Hospital of Western Theater Command, Chengdu, China; ^9^ Department of Medical Affairs, Air Force Hospital of Western Theater Command, Chengdu, China; ^10^ Center for Digestive Disease, The Seventh Affiliated Hospital, Sun Yat-sen University, Shenzhen, China; ^11^ Department of Digestive Diseases, the Second Affiliated Hospital of Chengdu Medical College, Chengdu, China; ^12^ Department of Digestive Diseases, the Affiliated Hospital of Southwest Medical University, Luzhou, China; ^13^ Department of Oncology, Qingdao Women and Children's Hospital, Qingdao, China; ^14^ Department of Emergency, Shaanxi Provincial People's Hospital, Xi’an, China; ^15^ Department of Digestive Diseases, Xi'an First Hospital, Xi’an, China; ^16^ Department of Digestive Diseases, Sanmenxia Central Hospital, Henan University of Science and Technology, Sanmenxia, China; ^17^ Department of Digestive Diseases, Daping Hospital, Army Medical University (Third Military Medical University), Chongqing, China; ^18^ Department of Surgery, Tangdu Hospital, Air Force Medical University (Fourth Military Medical University), Xi’an, China; ^19^ State Key Laboratory of Cancer Biology, Medical Genetics and Development Biology, Fourth Military Medical University, Xi’an, China; ^20^ Department of Digestive Diseases, General Hospital of Northern Theater Command, Shenyang, China; ^21^ Department of Digestive Diseases, Shanxi Bethune Hospital, Shanxi Academy of Medical Science, Tongji Shanxi Hospital, Third Hospital of Shanxi Medical University, Taiyuan, China; ^22^ Tongji Hospital, Tongji Medical College, Huazhong University of Science and Technology, Wuhan, China; ^23^ Department of Digestive Diseases, Tangdu Hospital, Air Force Medical University (Fourth Military Medical University), Xi’an, China

**Keywords:** hepatocellular carcinoma, transarterial chemoembolization, sorafenib, HAP series scores, predictive value

## Abstract

**Background:**

Hepatoma arterial-embolization prognostic (HAP) series scores have been proposed for prognostic prediction in patients with unresectable hepatocellular carcinoma (uHCC) undergoing transarterial chemoembolization (TACE). However, their prognostic value in TACE plus sorafenib (TACE-S) remains unknown. Here, we aim to evaluate their prognostic performance in such conditions and identify the best model for this combination therapy.

**Methods:**

Between January 2012 and December 2018, consecutive patients with uHCC receiving TACE-S were recruited from 15 tertiary hospitals in China. Cox regression analyses were used to investigate the prognostic values of baseline factors and every scoring system. Their prognostic performance and discriminatory performance were evaluated and confirmed in subgroup analyses.

**Results:**

A total of 404 patients were enrolled. In the whole cohort, the median follow-up period was 44.2 (interquartile range (IQR), 33.2–60.7) months, the median overall survival (OS) time was 13.2 months, and 336 (83.2%) patients died at the end of the follow-up period. According to multivariate analyses, HAP series scores were independent prognostic indicators of OS. In addition, the C-index, Akaike information criterion (AIC) values, and time-dependent area under the receiver operating characteristic (ROC) curve (AUC) indicated that modified HAP (mHAP)-III had the best predictive performance. Furthermore, the results remained consistent in most subsets of patients.

**Conclusion:**

HAP series scores exhibited good predictive ability in uHCC patients accepting TACE-S, and the mHAP-III score was found to be superior to the other HAP series scores in predicting OS. Future prospective high-quality studies should be conducted to confirm our results and help with treatment decision-making.

## Introduction

Transarterial chemoembolization (TACE) is the mainstay of therapy modalities for unresectable hepatocellular carcinoma (uHCC) patients in real-world clinical practice, while upregulation of vascular endothelial growth factor (VEGF) and platelet-derived growth factor (PDGF) receptor after TACE is closely associated with poor prognosis (1, 2). As a commonly used systematic treatment, sorafenib could suppress the factors mentioned above; thus, the treatment strategy of TACE plus sorafenib (TACE-S) is theoretically proposed to be a “good marriage” (3–5). Nevertheless, previous randomized controlled trials (RCTs) and observational studies have failed to reach a consensus on whether the combined use of sorafenib could bring survival benefits for uHCC patients as compared with TACE alone (6–12). Moreover, the median overall survival (OS) of uHCC patients undergoing TACE-S varies widely from 15.1 to 29.7 months (6–13). Therefore, we might infer that there was high heterogeneity among uHCC patients treated with TACE-S, and a well-performing prognostic model would be helpful for accurate survival prediction, as well as individual patient selection.

Unlike other solid tumors, liver function also plays an important role in decision-making and prognostic evaluation in addition to the tumor itself (14, 15). Child–Pugh classification, albumin–bilirubin (ALBI) grade, and platelet–albumin–bilirubin (PALBI) grade were used to assess liver function in clinical practice and were verified to be predictive for survival in uHCC patients treated with TACE-S (13, 16). Considering both tumor- and liver function-related factors, the hepatoma arterial-embolization prognostic (HAP) score (including tumor size, bilirubin, albumin, and α-fetoprotein (AFP)) has exhibited a promising prediction performance in uHCC patients following TACE (17–20). Subsequently, modified HAP (mHAP), mHAP-II, and mHAP-III scores were developed to enhance the prognostic ability of the HAP score originally proposed by L. Kadalayil. Nevertheless, the prognostic value of HAP series scores remained unknown in uHCC patients undergoing TACE-S.

In summary, this large multicenter exploratory study aims to investigate the prognostic factors in uHCC patients undergoing TACE-S, evaluate the predictive values of HAP series scores, and identify the most reliable one for survival prediction and patient selection.

## Materials and methods

### Study population and eligibility

**Figure 1:**
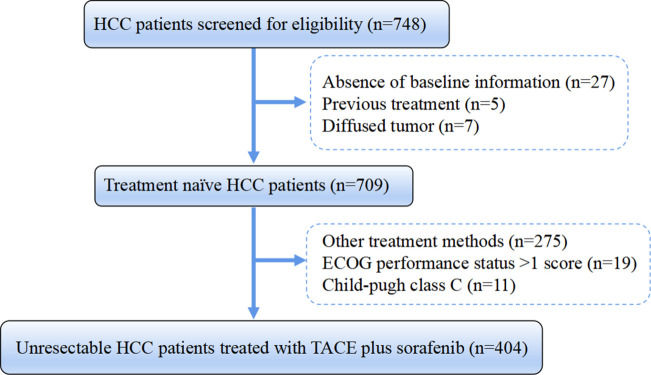
Flowchart of the patient selection process. HCC, hepatocellular carcinoma; HBV, hepatitis B virus; ECOG, Eastern Cooperative Oncology Group; TACE, transarterial chemoembolization.

Between January 2012 and December 2018, study data on consecutive uHCC patients receiving TACE-S were retrospectively extracted from a multicenter database of 15 Chinese tertiary hospitals. HCC was diagnosed according to the American Association for the Study of Liver Diseases/European Association for the Study of the Liver guidelines (21, 22). Patients needed to meet the following inclusion criteria: I) Child–Pugh grade A or B, II) Eastern Cooperative Oncology Group performance status (ECOG-PS) score of 0 or 1, III) time interval between the first TACE and sorafenib initiation at no more than 30 days, and IV) treatment-naïve uHCC patients. Patients were excluded based on the following criteria: I) missing variables included in calculating HAP, modified HAP (mHAP, mHAP-II, and mHAP-III) scores; II) combined with other tumors or severe cardiac, cerebral, and renal insufficiency; III) diffused tumor; IV) moderate or severe ascites. Finally, a total of 404 eligible HCC patients undergoing TACE-S were included (**Figure 1**). Written informed consent was obtained from all patients before treatment initiation, which consisted of consent to treatment and the potential use of clinical data in future investigations. The study protocol conformed to the ethical guidelines of the 1975 Declaration of Helsinki and was approved by the institute’s committee on human research of each participating center.

### Treatment and follow-up

According to the study protocol, treatment decisions were made at the discretion of the institutional multidisciplinary liver tumor boards of each enrolled center. Before TACE, hepatic arteriography was carried out to evaluate the vascular anatomy and tumor vascularity. During the TACE procedure, a vascular catheter was inserted selectively into the tumor-feeding artery with an injection containing a mixture of doxorubicin (10–50 mg) and lipiodol (2–20 ml), cisplatin (10–110 mg), epirubicin (10–50 mg), and oxaliplatin (100–200 mg), which were selected according to the practice of each center, followed by embolization using gelatin sponge particles. When residual viable tumors were confirmed or new lesions developed in patients with adequate liver function, repeated TACE was permitted. At an initial dose of 400 mg twice daily, sorafenib was initiated before/at/after the day of the first TACE and continuously used with no breaks thereafter. Moreover, the dose of sorafenib could be modified on the basis of the presence of toxicity and individuals’ drug tolerance. In general, patients were encouraged to continue sorafenib therapy unless unmanageable or life-threatening adverse events occurred. The patients who were concomitantly treated by sorafenib within 30 days before or after initial TACE were considered to be receiving TACE-S therapy. All patients were followed up at 1 month after TACE therapy and then at 3-month intervals in the first year and every 6–12 months thereafter. In clinical practice, the intensity of follow-up depends on individuals’ baseline characteristics and responses to the last treatment, as appropriate. Routine examinations were conducted at each follow-up, which included physical examinations, blood tests (tumor markers, blood and urine routine, and liver and renal function), and imaging examinations (chest X-ray, abdominal ultrasonography, abdominal contrast-enhanced CT, or MRI). The follow-up of the last patient was completed in September 2021.

### HAP serial score calculation

The detailed scoring method of each HAP series score is shown in **Table 1**. All HAP scores and their modified versions included the most significant indicators of OS: albumin, AFP, and tumor size. However, not exactly the same as the HAP score, mHAP removed the variable bilirubin; mHAP-II added tumor number based on the HAP score; and with the same factors of mHAP-II, the mHAP-III components were continuous instead of dichotomized (17–20).

**Table 1:** HAP serial score calculation.

Prognostic model	HAP (17)	mHAP (18)	mHAP-II (19)	mHAP-III (20)
Author (year)	L. Kadalayil et al. (2013)	David J. Pinato et al. (2015)	Yehyun Park et al. (2016)	Alberta Cappelli et al. (2016)
Sample size (n)	114	723	280	361
Prognostic factors	a. Albumin (<36 g/dl: 1 point; ≥36 g/dl: 0 points) b. Bilirubin (>17 μmol/L: 1 point; ≤17 μmol/L: 0 points) c. AFP (>400 ng/ml: 1 point; ≤400 ng/ml: 0 points) d. Tumor size (>7 cm: 1 point; ≤7 cm: 0 points)	a. Albumin (<36 g/dl: 1 point; ≥36 g/dl: 0 points) b. AFP (>400 ng/ml: 1 point; ≤400 ng/ml: 0 points) c. Tumor size (>7 cm: 1 point; ≤7 cm: 0 points)	a. Albumin (<36 g/dl: 1 point; ≥36 g/dl: 0 points) b. Bilirubin (>17 μmol/L: 1 point; ≤17 μmol/L: 0 points) c. AFP (>400 ng/ml: 1 point; ≤400 ng/ml: 0 points) d. Tumor size (>7 cm: 1 point; ≤7 cm: 0 points) e. Tumor number (≥2 nodules: 1 points; <2 nodules: 0 points)	(0.104 * size in cm) + (0.3089 * number (single nodule = 1; 2–3 nodules = 2; more than three nodules = 3)) + (0.2185 * Log10AFP in ng/ml) − (0.4049 * Albumin in g/dl) + (0.1506 * Bilirubin in mg/dl)
Classification	HAP A: 0; HAP B: 1; HAP C: 2; HAP D: >2	mHAP A: 0; mHAP B: 1; mHAP C: 2; mHAP D: >2	mHAP-II A: 0; mHAP-II B: 1; mHAP-II C: 2; mHAP-II D: >2	–

HAP, hepatoma arterial-embolization prognostic; mHAP, modified HAP; AFP, α-fetoprotein.

### Statistical analysis

Continuous variables were described by the mean with standard deviation (SD) or median with interquartile range (IQR). Categorical variables were expressed as frequencies and percentages. OS was defined as the time from the first session of TACE until death or last follow-up, and patients who were still alive were censored at the date of the last contact. Median OS was estimated using the Kaplan–Meier curves and compared with the log-rank test. Univariate and multivariate Cox proportional hazards regression models were used to analyze independent prognostic factors. Notably, five multivariate models with stepwise methods were separately conducted to avoid collinearity: model 1 included the baseline characteristics; model 2 included the baseline characteristics and HAP score but excluded albumin, AFP, tumor size, and bilirubin; model 3 included the baseline characteristics and mHAP score but excluded albumin, AFP, and tumor size; model 4 included the baseline characteristics and mHAP-II score but excluded albumin, AFP, bilirubin, tumor number, and tumor size; model 5 included the baseline characteristics and mHAP-III score but excluded albumin, AFP, bilirubin, tumor number, and tumor size. The discriminatory abilities of different prognostic score methods were compared using the C-index and time-dependent area under the receiver operating characteristic curve (AUC). Correlation analysis was performed by Kendall’s rank correlation coefficient tau-b. The Akaike information criterion (AIC) was also calculated to compare the loss of information for different models. The net reclassification improvement (NRI) statistic and the integrated discrimination improvement (IDI) statistic were used to evaluate the overall improvement in predictive value among HAP series scores. Subgroup analyses for the above evaluation indicators were conducted among different baseline backgrounds in order to avoid the potential influence of confounders. An additional benefit was also evaluated using decision curve analysis (DCA). Briefly, DCA was used to calculate the net benefit of new markers across various risk thresholds by taking into account weighted risks and benefits. Two-tailed p-values <0.05 for all analyses were identified as statistically significant. Statistical analyses were performed using R version 4.0.1 (R Foundation for Statistical Computing, Vienna, Austria) and IBM SPSS software version 26.0 (SPSS Inc., Chicago, IL, USA).

## Results

### Patient characteristics

Among the 404 enrolled patients, the mean age was 52.2 years, 336 (83.2%) patients were male, and the most common etiology was hepatitis B virus infection (337, 83.4%). The median tumor size (maximum diameter of the largest tumor) was 8.4 (IQR, 6.0–11.5) cm, and the median tumor number was 1 (IQR, 1.0–2.8). Additionally, 194 (48.0%) patients were classified as ECOG-PS of 0. Extrahepatic spread (EHS) was present in 12.9% (52), and portal vein tumor thrombosis (PVTT) was noted in 17.1% (69) of the whole population. According to the HAP, mHAP, and mHAP-II scoring systems, patients were divided into four distinct groups (A, B, C, and D). In addition, the median mHAP-III score of all patients was 0.50 (IQR, 0.03–1.07). For consistency with the scoring systems mentioned above, patients were classified into four groups (A, B, C, and D) based on the median and IQR of the mHAP-III score. The detailed baseline characteristics are described in **Table 2**.

**Table 2:** Baseline characteristics for the study patients (n = 404).

Characteristics	Values
Gender, male/female, n (%)	336 (83.2)/68 (6.8)
Age at start, year, mean ± SD	52.2 ± 12.6
Etiology, HBV/non-HBV, n (%)	337 (83.4)/67 (16.6)
Tumor size, cm, median (IQR)	8.4 (6.0–11.5)
Tumor number, cm, median (IQR)	1.0 (1.0–2.8)
PVTT, positive/negative, n (%)	69 (17.1)/335 (82.9)
EHS, positive/negative, n (%)	52 (12.9)/352 (87.1)
AFP, ≤400/>400 ng/ml, n (%)	212 (52.5)/192 (47.5)
HGB, g/L, mean ± SD	134.4 ± 21.7
PLT, 10^9^/L, median (IQR)	141.0 (89.0–188.5)
INR, median (IQR)	1.09 (1.02–1.19)
ALT, U/L, median (IQR)	37.5 (25.0–56.0)
AST, U/L, median (IQR)	48.5 (31.0–74.0)
ALB, g/L, mean ± SD	39.1 ± 50.2
TBIL, μmol/L, median (IQR)	15.4 (11.3–20.9)
BUN, mmol/L, median (IQR)	4.7 (3.9–5.7)
SCr, μmol/L, median (IQR)	81.0 (69.0–94.0)
Child–Pugh class, A/B, n (%)	368 (91.1)/36 (8.9)
Ascites, positive/negative, n (%)	50 (12.4)/354 (87.6)
ECOG score, 0/1, n (%)	194 (48.0)/210 (52.0)
BCLC stage, A/B/C/D, n (%)	88 (21.8)/65 (16.1)/201 (49.8)/50 (12.4)
TNM classification, I_B_/II/III_A_/IV_A_/IV_B_	149 (36.9)/65 (16.1)/138 (34.2)/7 (1.7)/45 (11.1)
HAP, A/B/C/D, n (%)	35 (8.7)/129 (31.9)/148 (36.6)/92 (22.8)
mHAP, A/B/C/D, n (%)	59 (14.6)/170 (42.1)/145 (35.9)/30 (7.4)
mHAP-II, A/B/C/D, n (%)	16 (4.0)/85 (21.0)/126 (31.2)/177 (43.8)
mHAP-III score, median (IQR)	0.5 (0.0–1.1)

SD, standard deviation; HBV, hepatitis B virus; IQR, interquartile range; AFP, α-fetoprotein; ECOG, Eastern Cooperative Oncology Group; BCLC, Barcelona Clinic Liver Cancer; PVTT, portal vein tumor thrombosis; EHS, extrahepatic spread; HAP, hepatoma arterial-embolization prognostic; mHAP, modified HAP; AST, aspartate aminotransferase; ALT, alanine aminotransferase; BUN, blood urea nitrogen; SCr, serum creatinine; INR, international normalized ratio; HGB, hemoglobin; TBIL, total bilirubin; ALB, albumin; PLT, platelet.

### Survival analysis of the whole cohort

**Figure 2:**
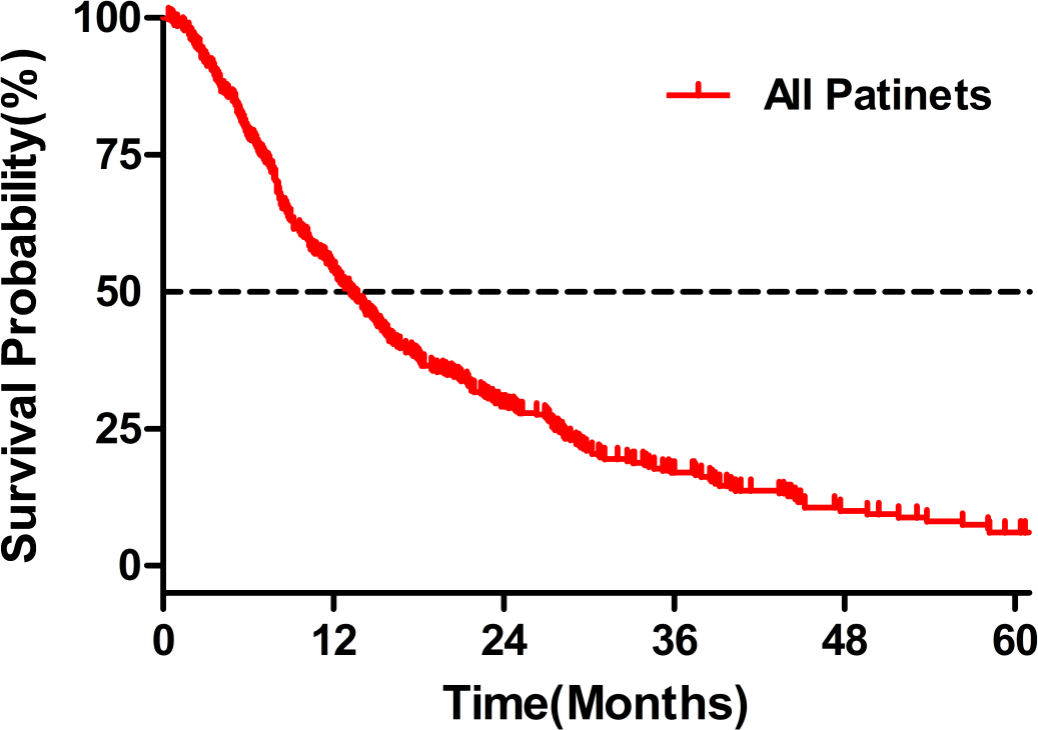
Survival analyses in the whole cohort.

In the whole cohort, the median follow-up period was 44.2 (IQR, 33.2–60.7) months, 68 (16.8%) patients were alive at the end of the follow-up period, and 336 (83.2%) patients had died. The median OS of the entire cohort reached 13.2 [95% confidence interval (CI) 11.6–14.8] months with 1-, 2-, and 3-year survival rates of 53.9%, 29.1%, and 17.0%, respectively (**Figure 2**). In univariate and multivariate analyses (**Tables 2**, **3**), tumor size (adjusted HR 1.047, 95% CI 1.012–1.085), tumor number (adjusted HR 1.040, 95% CI 1.091–1.191), AFP (adjusted HR 1.271, 95% CI 1.010–1.600), total bilirubin (TBIL) (adjusted HR 1.021, 95% CI 1.005–1.037), ALB (adjusted HR 0.952, 95% CI 0.927–0.977), PVTT (adjusted HR 3.020, 95% CI 2.202–4.142), and EHS (adjusted HR 2.082, 95% CI 1.503–2.886) were independent significant predictors of OS (all p < 0.05).

**Table 3:** Univariate analyses for OS in the whole cohort.

Characteristics	Univariate analyses	
	HR (95% CI)	p-Value
Gender, male (ref: female)	1.112 (0.831–1.489)	0.475
Age, per year increase	0.995 (0.987–1.004)	0.273
Etiology, others (ref: HBV)	0.834 (0.624–1.114)	0.218
Tumor size, per 1 cm increase	1.094 (1.064–1.125)	<0.001
Tumor number, per 1 lesion increase	1.180 (1.130–1.231)	<0.001
AFP > 400 ng/ml (ref: ≤400 ng/ml)	1.461 (1.178–1.810)	0.001
ALB, per 1 g/dl increase	0.938 (0.918–0.959)	<0.001
TBIL, per 1 μmol/L increase	1.030 (1.016–1.045)	<0.001
AST, per 1 U/L increase	1.007 (1.005–1.009)	<0.001
ALT, per 1 U/L increase	1.001 (0.998–1.004)	0.368
PLT, per 1 × 10^9^/L increase	1.001 (0.999–1.002)	0.376
INR, per 1% increase	2.357 (1.487–3.737)	<0.001
BUN, per 1 mmol/L increase	1.005 (0.932–1.083)	0.904
SCr, per 1 μmol/L increase	0.998 (0.982–0.995)	<0.001
Ascites, positive (ref: negative)	1.720 (1.263–2.343)	0.001
PVTT, positive (ref: negative)	3.593 (2.708–4.768)	<0.001
EHS, positive (ref: negative)	1.759 (1.302–2.378)	<0.001
ECOG, per 1 grade increase	2.245 (1.802–2.798)	<0.001
HAP score, per 1 grade increase	1.604 (1.414–1.819)	<0.001
mHAP score, per 1 grade increase	1.682 (1.466–1.930)	<0.001
mHAP-II score, per 1 grade increase	1.706 (1.488–1.956)	<0.001
mHAP-III score, per 1 score increase	2.319 (1.972–2.726)	<0.001

OS, overall survival; HR, hazard ratio; HBV, hepatitis B virus; AFP, α-fetoprotein; ECOG, Eastern Cooperative Oncology Group; PVTT, portal vein tumor thrombosis; EHS, extrahepatic spread; AST, aspartate aminotransferase; ALT, alanine aminotransferase; BUN, blood urea nitrogen; SCr, serum creatinine; INR, international normalized ratio; TBIL, total bilirubin; ALB, albumin; PLT, platelet.

### Prognostic values of HAP series scores in TACE-S

**Figure 3:**
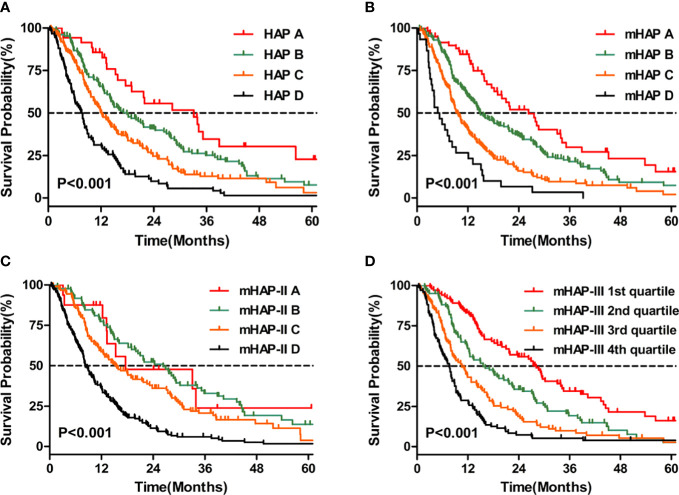
Kaplan–Meier curves for OS. **(A)** Comparison of survival between patients with HAP A, HAP B, HAP C, and HAP D **(B)** Comparison of survival between patients with mHAP A, mHAP B, mHAP C, and mHAP D **(C)** Comparison of survival between patients with mHAP-II A, mHAP-II B, mHAP-II C, and mHAP-II D **(D)** Comparison of survival between patients with mHAP-III (1st quartile, 2nd quartile, 3rd quartile, and 4th quartile). HAP, hepatoma arterial-embolization prognostic; mHAP, modified hepatoma arterial-embolization prognostic; OS, overall survival.

According to the Kaplan–Meier analyses, the HAP, mHAP, and mHAP-III scores had obvious discriminatory abilities among the A, B, C, and D groups (p < 0.05), whose OS showed a gradient downward trend (**Figures 3A, B, D** **)**. However, although mHAP-II had a gradient downward trend in median survival through classes, it could not distinguish patients between Groups A and B (p = 0.935) or between Groups A and C (p = 0.183) (**Figure 3C**). According to multivariate models 2 to 5, the HAP (adjusted HR 1.274, 95% CI 1.107–1.466), mHAP (adjusted HR 1.266, 95% CI 1.084–1.478), mHAP-II (adjusted HR 1.422, 95% CI 1.230–1.644), and mHAP-III (adjusted HR 1.772, 95% CI 1.455–2.158) score systems remained independent predictors of OS in patients treated with TACE-S (all p < 0.05, **Table 4**).

**Table 4:** Multivariate analyses for OS in the whole cohort.

Characteristics	Model 1		Model 2		Model 3		Model 4		Model 5	
	HR (95% CI)	P value	HR (95% CI)	P value	HR (95% CI)	P value	HR (95% CI)	P value	HR (95% CI)	P value
Tumor size, per 1 cm increase	1.047 (1.012–1.085)	0.009								
Tumor number, per 1 lesion increase	1.140 (1.091–1.191)	<0.001	1.140 (1.092–1.191)	<0.001	1.141 (1.093–1.192)	<0.001				
AFP > 400 ng/ml (ref: ≤400 ng/ml)	1.271 (1.010–1.600)	0.041								
ALB, per 1 g/dl increase	0.952 (0.927–0.977)	<0.001								
TBIL, per 1 μmol/L increase	1.021 (1.005–1.037)	0.008			1.017 (1.002–1.691)	0.029				
AST, per 1 U/L increase	1.002 (0.999–1.005)	0.119	1.004 (1.001–1.006)	0.004	1.003 (1.001–1.006)	0.010	1.004 (1.002–1.006)	0.001	1.002 (1.000–1.005)	0.106
INR, per 1% increase	1.202 (0.621–2.326)	0.585	1.428 (0.832–2.452)	0.196	1.381 (0.783–2.438)	0.265	1.558 (0.943–2.575)	0.083	1.726 (1.069–2.787)	0.025
SCr, per 1 μmol/L increase	0.999 (0.993–1.005)	0.742	0.996 (0.990–1.002)	0.178	0.997 (0.991–1.002)	0.251	0.995 (0.989–1.001)	0.098	0.998 (0.992–1.004)	0.457
Ascites, positive (ref: negative)	1.035 (0.731–1.463)	0.848	1.052 (0.747–1.482)	0.770	1.021 (0.722–1.443)	0.907	1.145 (0.819–1.602)	0.428	1.230 (0.879–1.720)	0.226
PVTT, positive (ref: negative)	3.020 (2.202–4.142)	<0.001	2.908 (2.134–3.962)	<0.001	2.923 (2.143–3.986)	<0.001	2.661 (1.952–3.626)	<0.001	2.539 (1.857–3.470)	<0.001
EHS, positive (ref: negative)	2.082 (1.503–2.886)	<0.001	2.007 (1.462–2.756)	<0.001	1.983 (1.445–2.722)	<0.001	2.045 (1.490–2.807)	<0.001	1.929 (1.403–2.650)	<0.001
ECOG, per 1 grade increase	1.201 (0.925–1.559)	0.170	1.340 (1.040–1.727)	0.023	1.306 (1.009–1.691)	0.042	1.426 (1.115–1.824)	0.005	1.290 (1.005–1.657)	0.046
HAP score, per 1 grade increase			1.274 (1.107–1.466)	0.001						
mHAP score, per 1 grade increase					1.266 (1.084–1.478)	0.003				
mHAP-II score, per 1 grade increase							1.422 (1.230–1.644)	<0.001		
mHAP-III score, per 1 score increase									1.772 (1.455–2.158)	<0.001

AFP, α-fetoprotein; ECOG, Eastern Cooperative Oncology Group; PVTT, portal vein tumor thrombosis; EHS, extrahepatic spread; HAP, hepatoma arterial-embolization prognostic; mHAP, modified HAP; AST, aspartate aminotransferase; SCr, serum creatinine; INR, international normalized ratio; TBIL, total bilirubin; ALB, albumin.

By comparing high-grade HAP series scores (grade C/D) with low-grade HAP series scores (grade A/B), there were significant differences in age, etiology, PVTT, and liver and renal function (platelet (PLT), aspartate aminotransferase (AST), alanine aminotransferase (ALT), blood urea nitrogen (BUN), and serum creatinine (SCr)) in addition to the components of HAP series scores (**Tables S1–4**). Furthermore, Kendall’s tau-b analysis showed that there were certain correlations among HAP series scores (**Figure S1**).

### Comparing the performance of HAP series scores

**Figure 4:**
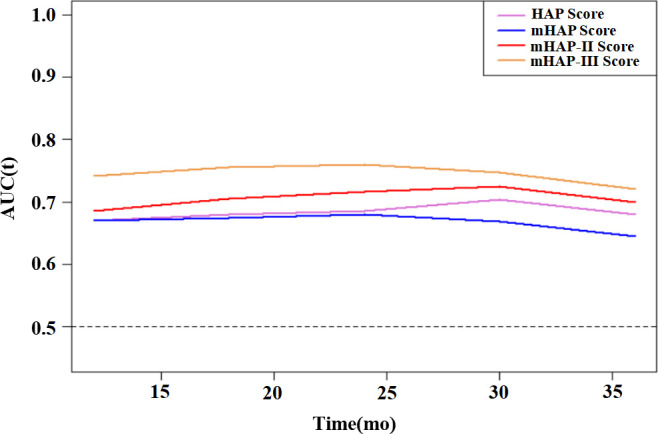
Time-dependent AUC for HAP series scores for predicting OS. HAP, hepatoma arterial-embolization prognostic; mHAP, modified hepatoma arterial-embolization prognostic; OS, overall survival; mo, months; AUC, area under the receiving operating curve.

**Figure 5:**
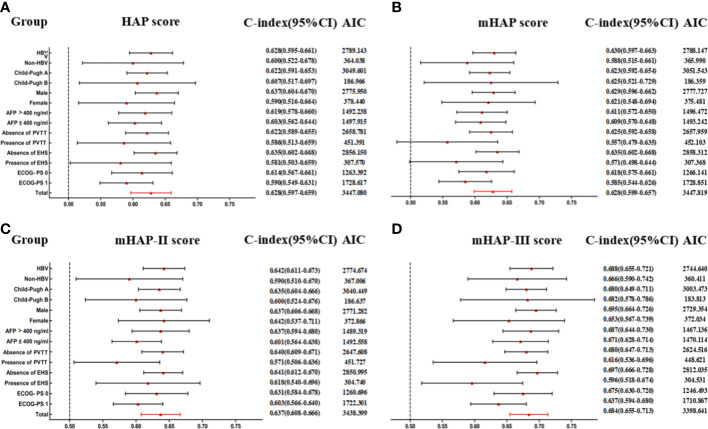
Subgroup analyses for HAP series scores to predict OS. **(A)** Predictive ability of HAP score in different subgroups. **(B)** Predictive ability of mHAP score in different subgroups. **(C)** Predictive ability of mHAP-II score in different subgroups. **(D)** Predictive ability of mHAP-III score in different subgroups. AIC, Akaike information criterion; HBV, hepatitis B virus; AFP, α-fetoprotein; ECOG, Eastern Cooperative Oncology Group; PS, performance status; HAP, hepatoma arterial-embolization prognostic; mHAP, modified HAP.

**Figure 6:**
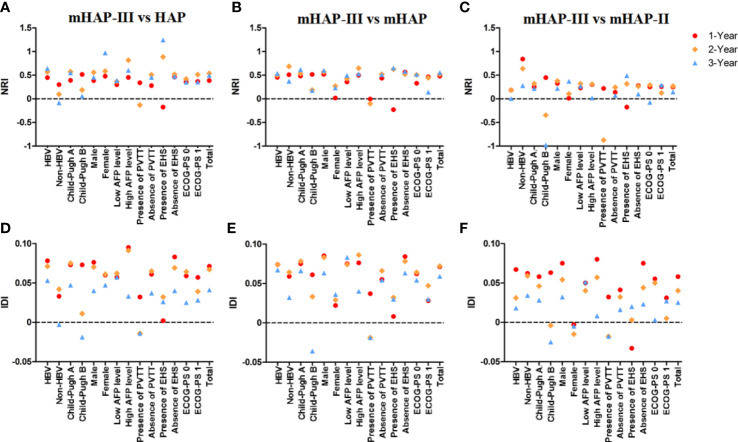
Net reclassification index (NRI) and integrated discrimination improvement (IDI) statistics. **(A–C)** NRI for mHAP-III *vs.* HAP, mHAP-III *vs.* mHAP, and mHAP-III *vs.* mHAP-II. **(D–F)** IDI for mHAP-III *vs.* HAP, mHAP-III *vs.* mHAP, and mHAP-III *vs.* mHAP-II. HAP, hepatoma arterial-embolization prognostic; mHAP, modified HAP.

**Figure 7:**
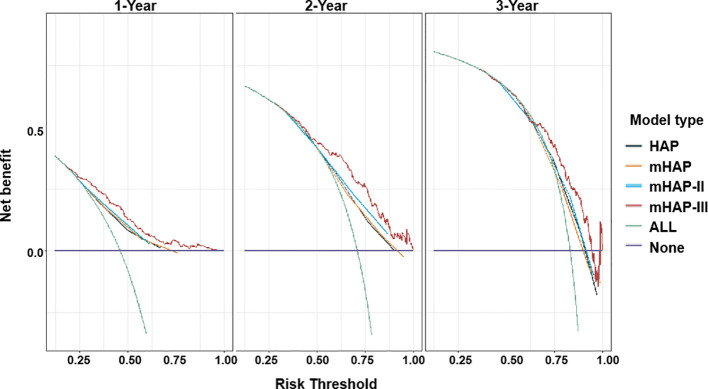
Decision curve analysis (DCA) for HAP series scores. Net benefit of using a model to predict 1-, 2-, and 3-year events of death as compared with strategies of “assume high risk to all” or “assume low risk to all” for different thresholds. HAP, hepatoma arterial-embolization prognostic.

On the basis of time-dependent AUC analysis and AIC value, mHAP-III had the lowest AIC value (C-index, 0.684; AIC, 3398.64), which indicated a more favorable prognostic performance and model-fitting ability as compared with the HAP (C-index, 0.628; AIC, 3447.08), mHAP (C-index, 0.628; AIC, 3447.82) and mHAP-II score (C-index, 0.637; AIC, 3438.40) in the whole cohort (p < 0.05) (**Figure 4**). As was shown in the forest plots, mHAP-III still showed an obvious and stable predictive performance among the majority of subsets (**Figure 5**). The detailed p-value of mHAP-III compared with HAP, mHAP, and mHAP-II scores in the whole cohort and each subset has been clarified in **Table S5**. Notably, according to the NRI and IDI statistics, the predictive ability of the mHAP-III was improved as compared with that of other scoring systems at the time point of 1, 2, and 3 years in the whole cohort. Similarly, the superiority of mHAP-III in predicting survival was subsequently confirmed in subset analyses (**Figure 6**). Moreover, the performance of the BCLC stage (C-index, 0.662; AIC, 3426.11) and TNM classification (C-index, 0.634; AIC, 3455.08) significantly lowered the mHAP-III, especially in the hepatitis B virus (HBV) subsets (**Table S5**). The DCA curve showed that the HAP series models achieved great clinical benefits (**Figure 7**).

## Discussion

TACE-S is usually used for the treatment of uHCC in clinical practice, but there are no suitable methods available for individual survival prediction. By comparing the predictive abilities of HAP series scores in uHCC patients treated by TACE-S, this nationwide multicenter retrospective observational study found that HAP series scores could predict survival in TACE-S and that mHAP-III had the best discriminatory performance. The advantages of our study lie in the multicenter nature and large sample size, as well as the first time to explore the prognostic values of HAP series scores in TACE-S.

It has been demonstrated that the TACE procedure might upregulate the expression of hypoxia-inducible factor-1α (HIF-1α) and then activate the proangiogenic factors VEGF and PDGF, which are associated with early tumor recurrence and poor prognosis of HCC (1, 2). Acting through selectively targeting VEGF and PDGF receptors, sorafenib plays a vital role in suppressing angiogenesis and exerts direct antitumor effects (4, 5). Therefore, combining TACE and sorafenib may be a good strategy for improving clinical outcomes (3). Previous studies have reported a median OS of 15.1–29.7 months in uHCC patients treated with TACE-S (6–13). However, the median OS of 13.2 months in our study was shorter, which was probably attributed to a higher proportion of patients with ECOG 1, PVTT, and/or EHS. The large variation in OS indicated substantial heterogeneity among uHCC patients undergoing TACE-S. Therefore, using effective baseline clinical features to identify optimal candidates who tend to benefit most from TACE-S is needed. Tumor burden is closely related to the prognosis of HCC patients. According to a previous study, tumor size and number increased, and the death risk was significantly increased for HCC patients treated by TACE-S (13). Similarly, our analyses suggested that tumor size and tumor number were independent prognostic risk factors among those patients. In addition, high serum AFP level has been identified as a biomarker for HCC associated with a more aggressive tumor phenotype and inferior outcomes after different treatment modalities in accordance with our statistical analyses (23). However, except for the indicators of tumor aggressiveness, it should be noted that the prognosis of HCC is more complicated than that of other solid malignant tumors, as most HCC patients have underlying liver diseases, such as liver cirrhosis, which is a major hurdle in prognosis assessment and patient management. As expected, a number of studies have identified indicators of liver function (ALBI, PALBI, and Child–Pugh grade) associated with the prognosis of patients undergoing TACE-S (13, 16). In the current study, albumin and total bilirubin were also deemed as independent prognostic factors. Given the aforementioned reasons, we should take both tumor characteristics and indicators of liver function into consideration when evaluating the prognosis of HCC patients.

The HAP score integrated tumor size, AFP, bilirubin, and albumin together, and the three modified HAP series scores (mHAP, mHAP-II, and mHAP-III) were subsequently developed through various adjustments subsequently (17). All of these scores, which were originally used to predict the outcomes of HCC patients after TACE, were also proven to have predictive abilities in TACE-S in this study (18–20). Furthermore, mHAP-III still had the best prognostic performance consistently at each time point, as displayed in the time-dependent AUC, which might be because of the following: i) mHAP-III included more indicators than the HAP and mHAP scores, ii) the use of continuous variables in the mHAP-III provided detailed information and individual predictions, and iii) mHAP-III applied different weights for each independent prognostic factor. Subgroup analyses were also conducted to verify the stability of our results, and mHAP-III showed the highest C-index and the lowest AIC value, particularly in patients with good baseline characteristics. The reason might be that HAP series scores were initially established in well-performing HCC patients treated with TACE alone. It was also suggested that the HAP series scores might be more suitable for the uHCC patients treated with TACE-S in the early and intermediate stages. Moreover, although mHAP-II and mHAP-III had the same variables, mHAP-II was less discriminative than mHAP-III, which may be due to categorical variables on arbitrary or optimal cutoffs being used in mHAP-II, and appropriate weights were not designated in each enrolled variable. In aggregate, mHAP-III showed superior predictive accuracy and discriminatory abilities in patients treated with not only TACE but also TACE-S. In fact, the factors included in the HAP series are closely related to the prognosis of HCC patients, and they can also be used to predict the prognosis of HCC patients treated with other therapies.

To the best of our knowledge, PVTT and EHS reflect the aggressiveness of HCC and have been deemed as negative prognostic predictors in different staging systems (24–27). Beyond the guideline recommendation, TACE-S has been widely used to manage uHCC patients with PVTT and EHS in real-world clinical practice. In the present study, patients with PVTT (adjusted HR 3.020, 95% CI 2.202–4.142) and EHS (adjusted HR 2.082, 95% CI 1.503–2.886) were involved, increasing mortality risk by approximately two- to threefold in uHCC patients undergoing TACE-S. Therefore, considering that sorafenib is a systematic treatment for advanced HCC, a possible way would be to include factors such as both of them to further refine the prognostic model. Additionally, NRI was closely related to the set time point, and the survival time of patients with PVTT in this study was less than 3 years. Thus, there was no point in the presence of PVTT at 3 years (**Figures 6A–C**). Furthermore, ECOG-PS has also been identified as being associated with survival, which plays an important role in risk stratification for HCC patients (28). However, the influence of ECOG-PS did not reach significant statistical significance in model 1, which might be because ECOG-PS was affected by tumor burden and liver function, while the effect was offset by these cofounders. This finding emphasized from another perspective that huge heterogeneity exists in uHCC patients and individual patient-level prognostication should be conducted. Moreover, future studies could explore and consider multiple risk factors, such as age, etiology, and renal function, and integrate various evaluation indicators to find the optimal prediction model.

The results of this study should nevertheless be interpreted in light of several limitations. On the one hand, the existence of information bias in this article is inevitable due to its retrospective nature. To minimize potential bias, uHCC patients treated with TACE-S from a national multicenter were included, and multiple follow-up visits were attempted for each unreachable patient. Due to the decrease in sample size in each risk stratification, the statistical power was weakened during subgroup analysis. Consequently, a larger sample size and prospective research are needed to further verify the results of our study. Moreover, the retrospective study cannot explore the causal relationship between survival and the scoring system. We also will further explore this issue in subsequent prospective studies. On the other hand, most of the patients in our study had HBV-related HCC. In addition, hepatitis C virus infection and alcoholic liver disease are also important pathogenic factors of HCC (29, 30). It is worth noting that there has been a marked increase in non-viral hepatitis mostly caused by metabolic-associated fatty liver disease (MAFLD), gradually becoming one of the most critical medical issues in the field of hepatology (31). The generalization and application of our findings should be done with caution, and future prospective studies are needed. Last but not least, it is notable that macoscopic vascular invasion (MVI), EHS, and ECOG were independent risk factors associated with poor prognosis, and future studies should take these factors into consideration and assign weights appropriately to achieve individualized and accurate prediction for patients receiving TACE-S.

## Conclusion

In summary, we demonstrated that the HAP series scores exhibited good predictive ability in uHCC patients accepting TACE-S, and the mHAP-III score was found to be superior to the other HAP series scores in predicting OS. Future prospective high-quality studies should be conducted to confirm our results and help with treatment decision-making.

## Data availability statement

The raw data supporting the conclusions of this article will be made available by the authors, without undue reservation.

## Ethics statement

Written informed consent was obtained from the individual(s) for the publication of any potentially identifiable images or data included in this article.

## Author contributions

JL, XLD, DFC and LL conceived and supervised the project. YJZ, EXW, SJZ and DDH retrieved patients’ clinical information and conducted the telephone follow-up. YJZ performed data curation and analysis and prepared data visualization. YJZ, ENW, SJZ and DDH drafted the manuscript. JL, XLD, DFC and LL reviewed and revised the manuscript. YZ, HC, JZ, THH, YB, YJL, YCZ, MY, LZ, JHF, XC, JJ, WBW, WRR, TTB, SZM, FHX, YXT, YH, JLZ and XSQ provided patient care and performed the clinical assessments. All authors contributed to the article and approved the submitted version.

## Glossary

**Table:**

AFP	α-fetoprotein
AIC	Akaike information criterion
ALBI	albumin–bilirusbin
ALT	alanine aminotransferase
AST	aspartate aminotransferase
AUC	area under ROC curve
BCLC	Barcelona Clinic Liver Cancer
BUN	blood urea nitrogen
CI	confidence interval
CT	computed tomography
ECOG	Eastern Cooperative Oncology Group
EHS	extrahepatic spread
HCC	hepatocellular carcinoma
HAP	hepatoma arterial-embolization prognostic
HBV	hepatitis B virus
HCV	hepatitis C virus
HR	hazard ratio
IDI	integrated discrimination improvement
INR	international normalized ratio
IQR	interquartile range
MAFLD	metabolic-associated fatty liver disease
mHAP	modified HAP
MRI	magnetic resonance imaging
NRI	net reclassification improvement
OS	overall survival
pALBI	platelet–albumin–bilirubin
PDGF	platelet-derived growth factor
PS	performance status
PVTT	portal vein tumor thrombosis
RCT	randomized controlled trial
ROC	receiver operating characteristic
SD	standard deviation
TACE	transarterial chemoembolization
TBIL	total bilirubin
TKIs	multikinase inhibitor tyrosine kinase inhibitors
VEGF	vascular endothelial growth factor
