# Supplementary material for: Identify optimal HAP series scores for unresectable HCC patients undergoing TACE plus sorafenib: A Chinese multicenter observational study
Source: Front Oncol. 2023 Jan 27;12:983554. doi: 10.3389/fonc.2022.983554 (PMC9911813; doi:10.3389/fonc.2022.983554)

**Supplementary materials**

**Identify optimal HAP series scores for unresectable HCC patients undergoing TACE plus sorafenib: A Chinese Multicenter Observational Study**

Yejing Zhu, Enxin Wang, Shoujie Zhao, Dandan Han, Yan Zhao, Hui Chen, Jun Zhu, Tenghui Han, Yang Bai, Yanju Lou, Yongchao Zhang, Man Yang, Luo Zuo, Jiahao Fan, Xing Chen, Jia Jia, Wenbin Wu, Weirong Ren, Tingting Bai, Shouzheng Ma, Fenghua Xu, Yuxin Tang, Ying Han, Junlong Zhao, Xingshun Qi, Jing Li, Xilin Du, Dongfeng Chen, Lei Liu

| **Characteristics** | **HAP A/B** | **HAP C/D** | ***P value*** |
| --- | --- | --- | --- |
| Gender, male/female, n (%) | 138(84.1)/26(15.9) | 198(82.5)/42(17.5) | 0.664 |
| Age at start, year, mean±SD | 54.9±13.0 | 50.3±12.0 | <0.001 |
| Etiology, HBV/non-HBV, n (%) | 126 (76.8)/38 (23.3) | 211 (87.9)/29 (12.1) | 0.003 |
| Tumor size, cm, median (IQR) | 6.5 (5.0-9.6) | 9.8 (7.2-12.5) | <0.001 |
| Tumor number, cm, median (IQR) | 1.0 (1.0-2.0) | 2.0 (1.0-3.0) | 0.011 |
| PVTT, positive/negative, n (%) | 18 (11.0)/146 (89.0) | 51 (21.3)/189 (78.8) | 0.007 |
| EHS, positive/negative, n (%) | 20 (12.2)/144 (87.8) | 32 (13.3)/208 (86.7) | 0.737 |
| AFP, ≤ 400/> 400 ng/mL, n (%) | 134 (81.7)/30 (18.3) | 78 (32.5)/162 (67.5) | <0.001 |
| HGB, g/L, mean ± SD | 136.0±18.7 | 133.3±23.5 | 0.204 |
| PLT, 10^9/L, median (IQR) | 137.5 (93.0-186.0) | 142.5 (86.3-194.0) | 0.750 |
| INR, median (IQR) | 1.1 (1.0-1.1) | 1.1 (1.0-1.2) | 0.002 |
| ALT, U/L, median (IQR) | 35.0 (23.0-49.5) | 39.5 (27.0-58.8) | 0.013 |
| AST, U/L, median (IQR) | 36.0 (28.0-55.0) | 57.0 (37.3-89.5) | <0.001 |
| ALB, g/L, mean ± SD | 40.8±4.3 | 38.0±5.4 | <0.001 |
| TBIL, μmol/L, median (IQR) | 12.7 (9.7-15.8) | 18.7 (12.8-24.4) | <0.001 |
| BUN, mmol/L, median (IQR) | 5.0 (3.9-5.8) | 4.6 (3.8-5.6) | 0.019 |
| SCr, umol/L, median (IQR) | 84.0 (73.3-97.0) | 77.0 (67.0-92.0) | 0.001 |
| Ascites, positive/negtive, n (%) | 10 (6.1)/154 (93.9) | 40 (16.7)/200 (83.3) | 0.002 |
| ECOG score, 0/1, n (%) | 107 (65.2)/57 (34.8) | 87 (36.3)/153 (63.7) | <0.001 |

**Table S1.** Comparison of baseline characteristics between HAP A/B and HAP C/D

SD, standard deviation; HBV, hepatitis B virus; IQR, interquartile range; AFP, alphafetoprotein; ECOG, Eastern Cooperative Oncology Group; PVTT, portal vein tumor thrombosis; EHS, extrahepatic spread; HAP, hepatoma arterial-embolisation prognostic; mHAP, modified HAP; AST, aspartate aminotransferase; ALT, alanine aminotransferase; BUN, blood urea nitrogen; SCr, serum creatinine; INR, international normalized ratio; HGB, hemoglobin; TBIL, total bilirubin; ALB, albumin; PLT, platelets.

**Table S2.** Comparison of baseline characteristics between mHAP A/B and mHAP C/D

| **Characteristics** | **mHAP A/B** | **mHAP C/D** | ***P value*** |
| --- | --- | --- | --- |
| Gender, male/female, n (%) | 192 (83.8)/37 (54.4) | 144 (82.3)/31 (17.7) | 0.679 |
| Age at start, year, mean±SD | 54.8±12.7 | 48.9±11.7 | <0.001 |
| Etiology, HBV/non-HBV, n (%) | 180 (78.6)/49 (21.4) | 157 (89.7)/18 (10.3) | 0.003 |
| Tumor size, cm, median (IQR) | 6.5 (4.9-9.5) | 10.8 (8.4-13.1) | <0.001 |
| Tumor number, cm, median (IQR) | 1.0 (1.0-2.0) | 2.0 (1.0-3.0) | 0.017 |
| PVTT, positive/negative, n (%) | 24 (10.5)/205 (89.5) | 45 (25.7)/130 (74.3) | <0.001 |
| EHS, positive/negative, n (%) | 28 (12.2)/201 (87.8) | 24 (13.7)/151 (86.3) | 0.658 |
| AFP, ≤ 400/> 400 ng/mL, n (%) | 184 (80.3)/45 (19.7) | 28 (16.0)/147 (84.0) | <0.001 |
| HGB, g/L, mean ± SD | 135.6±19.4 | 132.9±24.3 | 0.225 |
| PLT, 10^9/L, median (IQR) | 126.0 (85.5-174.5) | 152.0 (97.0-207.0) | <0.001 |
| INR, median (IQR) | 1.1 (1.0-1.2) | 1.1 (1.0-1.2) | 0.201 |
| ALT, U/L, median (IQR) | 35.0 (23.0-52.0) | 42.0 (29.0-60.0) | 0.002 |
| AST, U/L, median (IQR) | 36.0 (28.0-56.0) | 64.0 (45.0-97.0) | <0.001 |
| ALB, g/L, mean ± SD | 40.2±5.1 | 37.7±5.0 | <0.001 |
| TBIL, μmol/L, median (IQR) | 14.8 (11.1-20.4) | 15.6 (11.5-21.9) | 0.289 |
| BUN, mmol/L, median (IQR) | 4.9 (4.0-5.8) | 4.5 (3.7-5.4) | 0.003 |
| SCr, umol/L, median (IQR) | 83.0 (71.0-96.0) | 77.0 (67.0-90.0) | 0.002 |
| Ascites, positive/negtive, n (%) | 20 (8.7)/209 (91.3) | 30 (17.1)/145 (82.9) | 0.011 |
| ECOG score, 0/1, n (%) | 142 (62.0)/87 (38.0) | 52 (29.7)/123 (70.3) | <0.001 |

SD, standard deviation; HBV, hepatitis B virus; IQR, interquartile range; AFP, alphafetoprotein; ECOG, Eastern Cooperative Oncology Group; PVTT, portal vein tumor thrombosis; EHS, extrahepatic spread; HAP, hepatoma arterial-embolisation prognostic; mHAP, modified HAP; AST, aspartate aminotransferase; ALT, alanine aminotransferase; BUN, blood urea nitrogen; SCr, serum creatinine; INR, international normalized ratio; HGB, hemoglobin; TBIL, total bilirubin; ALB, albumin; PLT, platelets.

| **Characteristics** | **mHAP-II A/B** | **mHAP-II C/D** | ***P value*** |
| --- | --- | --- | --- |
| Gender, male/female, n (%) | 83 (82.2)/18 (17.8) | 253 (83.5)/50 (16.5) | 0.759 |
| Age at start, year, mean±SD | 55.4±12.1 | 51.1±12.6 | 0.003 |
| Etiology, HBV/non-HBV, n (%) | 73 (72.3)/28 (27.7) | 264 (87.1)/39 (12.9) | 0.001 |
| Tumor size, cm, median (IQR) | 6.3 (4.3-8.5) | 9.2 (6.8-12.2) | <0.001 |
| Tumor number, cm, median (IQR) | 1.0 (1.0-1.0) | 2.0 (1.0-3.0) | <0.001 |
| PVTT, positive/negative, n (%) | 8 (7.9)/93 (92.1) | 61 (20.1)/242 (79.9) | 0.005 |
| EHS, positive/negative, n (%) | 13 (12.9)/88 (87.1) | 39 (12.9)/264 (87.1) | 1.000 |
| AFP, ≤ 400/> 400 ng/mL, n (%) | 88 (87.1)/13 (12.9) | 124 (40.9)/179 (59.1) | <0.001 |
| HGB, g/L, mean ± SD | 135.9±19.3 | 133.9±22.5 | 0.408 |
| PLT, 10^9/L, median (IQR) | 151.0 (95.0-189.5) | 141.0 (88.0-186.0) | 0.536 |
| INR, median (IQR) | 1.1 (1.0-1.1) | 1.1 (1.1-1.2) | 0.011 |
| ALT, U/L, median (IQR) | 35.0 (24.0-47.5) | 38.0 (26.0-58.0) | 0.036 |
| AST, U/L, median (IQR) | 35.0 (25.0-48.5) | 55.0 (35.0-84.0) | <0.001 |
| ALB, g/L, mean ± SD | 41.1±4.6 | 38.5±5.2 | <0.001 |
| TBIL, μmol/L, median (IQR) | 12.3 (9.6-14.6) | 17.2 (12.1-22.4) | <0.001 |
| BUN, mmol/L, median (IQR) | 5.1 (4.3-6.0) | 4.6 (3.8-5.6) | 0.014 |
| SCr, umol/L, median (IQR) | 84.0 (74.0-100.0) | 79.0 (68.0-92.0) | 0.002 |
| Ascites, positive/negtive, n (%) | 7 (6.9)/94 (93.1) | 43 (14.2)/260 (85.8) | 0.055 |
| ECOG score, 0/1, n (%) | 70 (69.3)/31 (30.7) | 124 (40.9)/179 (59.1) | <0.001 |

**Table S3.** Comparison of baseline characteristics between mHAP-II A/B and mHAP-II C/D

SD, standard deviation; HBV, hepatitis B virus; IQR, interquartile range; AFP, alphafetoprotein; ECOG, Eastern Cooperative Oncology Group; PVTT, portal vein tumor thrombosis; EHS, extrahepatic spread; HAP, hepatoma arterial-embolisation prognostic; mHAP, modified HAP; AST, aspartate aminotransferase; ALT, alanine aminotransferase; BUN, blood urea nitrogen; SCr, serum creatinine; INR, international normalized ratio; HGB, hemoglobin; TBIL, total bilirubin; ALB, albumin; PLT, platelets.

| **Characteristics** | **mHAP-III A/B** | **mHAP-III C/D** | ***P value*** |
| --- | --- | --- | --- |
| Gender, male/female, n (%) | 169 (83.7)/33 (16.3) | 167 (82.7)/35 (17.3) | 0.790 |
| Age at start, year, mean±SD | 54.7±13.1 | 49.7±11.5 | <0.001 |
| Etiology, HBV/non-HBV, n (%) | 154 (76.2)/48 (23.8) | 183 (90.6%)/19 (9.4) | <0.001 |
| Tumor size, cm, median (IQR) | 6.3 (4.3-8.5) | 11.0 (8.3-13.5) | <0.001 |
| Tumor number, cm, median (IQR) | 1.0 (1.0-1.0) | 2.0 (1.0-4.0) | <0.001 |
| PVTT, positive/negative, n (%) | 20 (9.9)/182 (90.1) | 49 (24.3)/153 (75.7) | <0.001 |
| EHS, positive/negative, n (%) | 24 (11.9)/178 (88.1) | 28 (13.9)/174 (86.1) | 0.552 |
| AFP, ≤ 400/> 400 ng/mL, n (%) | 152 (75.2)/50 (24.8) | 60 (29.7)/142 (70.3) | <0.001 |
| HGB, g/L, mean ± SD | 136.3±20.1 | 132.5±23.1 | 0.081 |
| PLT, 10^9/L, median (IQR) | 151.0 (95.0-189.5) | 150.0 (97.8-210.3) | <0.001 |
| INR, median (IQR) | 1.1 (1.0-1.1) | 1.1 (1.0-1.2) | 0.206 |
| ALT, U/L, median (IQR) | 35.0 (24.0-47.5) | 41.5 (29.0-60.3) | <0.001 |
| AST, U/L, median (IQR) | 35.0 (25.0-48.5) | 66.0 (45.0-98.0) | <0.001 |
| ALB, g/L, mean ± SD | 40.6±5.1 | 37.6±4.8 | <0.001 |
| TBIL, μmol/L, median (IQR) | 12.3 (9.6-14.6) | 15.9 (11.4-22.3) | 0.045 |
| BUN, mmol/L, median (IQR) | 5.1 (4.3-6.0) | 4.5 (3.7-5.3) | 0.001 |
| SCr, umol/L, median (IQR) | 84.0 (74.0-100.0) | 77.0 (67.0-90.0) | <0.001 |
| Ascites, positive/negtive, n (%) | 19 (9.4)/183 (90.6) | 31 (15.3)/171 (84.7) | 0.070 |
| ECOG score, 0/1, n (%) | 133 (65.8)/69 (34.2) | 61 (30.2)/141 (69.8) | <0.001 |

**Table S4.** Comparison of baseline characteristics between mHAP-III A/B and mHAP-III C/D

SD, standard deviation; HBV, hepatitis B virus; IQR, interquartile range; AFP, alphafetoprotein; ECOG, Eastern Cooperative Oncology Group; PVTT, portal vein tumor thrombosis; EHS, extrahepatic spread; HAP, hepatoma arterial-embolisation prognostic; mHAP, modified HAP; AST, aspartate aminotransferase; ALT, alanine aminotransferase; BUN, blood urea nitrogen; SCr, serum creatinine; INR, international normalized ratio; HGB, hemoglobin; TBIL, total bilirubin; ALB, albumin; PLT, platelets.

**Table S5.** Comparison of the performance of mHAP-III with HAP, mHAP, mHAP-II score，BCLC stage and TNM classification.

| **Population** | **△C-index** | **95% CI** | **P value** |
| --- | --- | --- | --- |
| **The Whole Cohort** |  |  |  |
| mHAP-III vs. mHAP-II | 0.047 | 0.023-0.073 | P<0.001 |
| mHAP-III vs. mHAP | 0.057 | 0.039-0.086 | P<0.001 |
| mHAP-III vs. HAP | 0.056 | 0.034-0.081 | P<0.001 |
| mHAP-III vs. BCLC | 0.022 | -0.016-0.059 | P=0.254 |
| mHAP-III vs. TNM | 0.050 | 0.004-0.101 | P=0.042 |
| **HBV(+) cohort** |  |  |  |
| mHAP-III vs. mHAP-II | 0.046 | 0.013-0.080 | P=0.008 |
| mHAP-III vs. mHAP | 0.058 | 0.037-0.076 | P<0.001 |
| mHAP-III vs. HAP | 0.060 | 0.029-0.086 | P<0.001 |
| mHAP-III vs. BCLC | 0.039 | 0.004-0.076 | P=0.033 |
| mHAP-III vs. TNM | 0.122 | 0.062-0.170 | P<0.001 |
| **HBV(-) cohort** |  |  |  |
| mHAP-III vs. mHAP-II | 0.076 | 0.027-0.118 | P=0.001 |
| mHAP-III vs. mHAP | 0.078 | 0.007-0.150 | P=0.033 |
| mHAP-III vs. HAP | 0.066 | -0.000-0.130 | P=0.046 |
| mHAP-III vs. BCLC | -0.051 | -0.169-0.045 | P=0.351 |
| mHAP-III vs. TNM | 0.033 | -0.113-0.170 | P=0.651 |
| **Chid-Pugh A cohort** |  |  |  |
| mHAP-III vs. mHAP-II | 0.045 | 0.023-0.062 | P<0.001 |
| mHAP-III vs. mHAP | 0.058 | 0.029-0.083 | P<0.001 |
| mHAP-III vs. HAP | 0.058 | 0.031-0.079 | P<0.001 |
| mHAP-III vs. BCLC | 0.016 | -0.019-0.055 | P=0.391 |
| mHAP-III vs. TNM | 0.049 | 0.009-0.084 | P=0.010 |
| **Child-Pugh B cohort** |  |  |  |
| mHAP-III vs. mHAP-II | 0.082 | -0.038-0.178 | P=0.138 |
| mHAP-III vs. mHAP | 0.056 | -0.052-0.141 | P=0.254 |
| mHAP-III vs. HAP | 0.074 | -0.019-0.141 | P=0.067 |
| mHAP-III vs. BCLC | 0.174 | 0.046-0.285 | P=0.004 |
| mHAP-III vs. TNM | 0.039 | -0.100-0.150 | P=0.545 |
| **Male cohort** |  |  |  |
| mHAP-III vs. mHAP-II | 0.057 | 0.037-0.072 | P<0.001 |
| mHAP-III vs. mHAP | 0.065 | 0.041-0.085 | P<0.001 |
| mHAP-III vs. HAP | 0.057 | 0.036-0.070 | P<0.001 |
| mHAP-III vs. BCLC | 0.033 | -0.009-0.060 | P=0.060 |
| mHAP-III vs. TNM | 0.068 | 0.021-0.098 | P=0.001 |
| **Female cohort** |  |  |  |
| mHAP-III vs. mHAP-II | 0.011 | -0.063-0.083 | P=0.769 |
| mHAP-III vs. mHAP | 0.032 | -0.028-0.071 | P=0.210 |
| mHAP-III vs. HAP | 0.063 | -0.009-0.128 | P=0.071 |
| mHAP-III vs. BCLC | -0.014 | -0.107-0.083 | P=0.778 |
| mHAP-III vs. TNM | 0.000 | -0.242-0.197 | P=0.997 |
| **AFP≤400 cohort** |  |  |  |
| mHAP-III vs. mHAP-II | 0.051 | 0.011-0.091 | P=0.012 |
| mHAP-III vs. mHAP | 0.076 | 0.044-0.123 | P<0.001 |
| mHAP-III vs. HAP | 0.069 | 0.018-0.135 | P=0.021 |
| mHAP-III vs. BCLC | -0.004 | -0.051-0.043 | P=0.861 |
| mHAP-III vs. TNM | 0.048 | -0.009-0.097 | P=0.078 |
| **AFP＞400 cohort** |  |  |  |
| mHAP-III vs. mHAP-II | 0.071 | 0.045-0.101 | P<0.001 |
| mHAP-III vs. mHAP | 0062 | 0.020-0.096 | P=0.001 |
| mHAP-III vs. HAP | 0.068 | 0.041-0.106 | P<0.001 |
| mHAP-III vs. BCLC | 0.045 | 0.001-0.098 | P=0.068 |
| mHAP-III vs. TNM | 0.048 | 0.010-0.091 | P=0.020 |
| **PVTT (+) cohort** |  |  |  |
| mHAP-III vs. mHAP-II | 0.045 | -0.051-0.112 | P=0.281 |
| mHAP-III vs. mHAP | 0.059 | -0.045-0.148 | P=0.230 |
| mHAP-III vs. HAP | 0.030 | -0.055-0.100 | P=0.445 |
| mHAP-III vs. BCLC | 0.058 | -0.030-0.158 | P=0.229 |
| mHAP-III vs. TNM | 0.151 | 0.051-0.324 | P=0.030 |
| **PVTT (-) cohort** |  |  |  |
| mHAP-III vs. mHAP-II | 0.040 | 0.011-0.071 | P=0.010 |
| mHAP-III vs. mHAP | 0.054 | 0.028-0.086 | P<0.001 |
| mHAP-III vs. HAP | 0.058 | 0.025-0.656 | P<0.001 |
| mHAP-III vs. BCLC | 0.029 | 0.003-0.056 | P=0.034 |
| mHAP-III vs. TNM | 0.042 | -0.000-0.078 | P=0.034 |
| **EHS (+) cohort** |  |  |  |
| mHAP-III vs. mHAP-II | -0.022 | -0.085-0.037 | P=0.477 |
| mHAP-III vs. mHAP | 0.024 | -0.043-0.112 | P=0.537 |
| mHAP-III vs. HAP | 0.015 | -0.044-0.090 | P=0.665 |
| mHAP-III vs. BCLC | 0.114 | 0.050-0.185 | P=0.001 |
| mHAP-III vs. TNM | 0.023 | -0.055-0.103 | P=0.574 |
| **EHS (-) cohort** |  |  |  |
| mHAP-III vs. mHAP-II | 0.056 | 0.029-0.092 | P=0.001 |
| mHAP-III vs. mHAP | 0.062 | 0.039-0.085 | P<0.001 |
| mHAP-III vs. HAP | 0.062 | 0.034-0.092 | P<0.001 |
| mHAP-III vs. BCLC | 0.000 | -0.079-0.070 | P=0.995 |
| mHAP-III vs. TNM | 0.052 | -0.032-0.122 | P=0.187 |
| **ECOG PS 0 cohort** |  |  |  |
| mHAP-III vs. mHAP-II | 0.044 | 0.016-0.084 | P=0.012 |
| mHAP-III vs. mHAP | 0.057 | 0.026-0.093 | P=0.001 |
| mHAP-III vs. HAP | 0.061 | 0.024-0.106 | P=0.004 |
| mHAP-III vs. BCLC | 0.000 | -0.079-0.070 | P=0.995 |
| mHAP-III vs. TNM | 0.059 | -0.191-0.208 | P=0.560 |
| **ECOG PS 1 cohort** |  |  |  |
| mHAP-III vs. mHAP-II | 0.035 | 0.005-0.074 | P=0.048 |
| mHAP-III vs. mHAP | 0.052 | 0.015-0.086 | P=0.004 |
| mHAP-III vs. HAP | 0.047 | 0.024-0.081 | P=0.001 |
| mHAP-III vs. BCLC | 0.114 | 0.050-0.185 | P=0.001 |
| mHAP-III vs. TNM | 0.023 | -0.055-0.103 | P=0.574 |

HBV, hepatitis B virus; AFP, alphafetoprotein; ECOG, Eastern Cooperative Oncology Group; PVTT, portal vein tumor thrombosis; EHS, extrahepatic spread; HAP, hepatoma arterial-embolisation prognostic; mHAP, modified HAP.

**Figure S1.** Correlations of HAP series scores. HAP, hepatoma arterial-embolisation prognostic; mHAP, modified HAP;


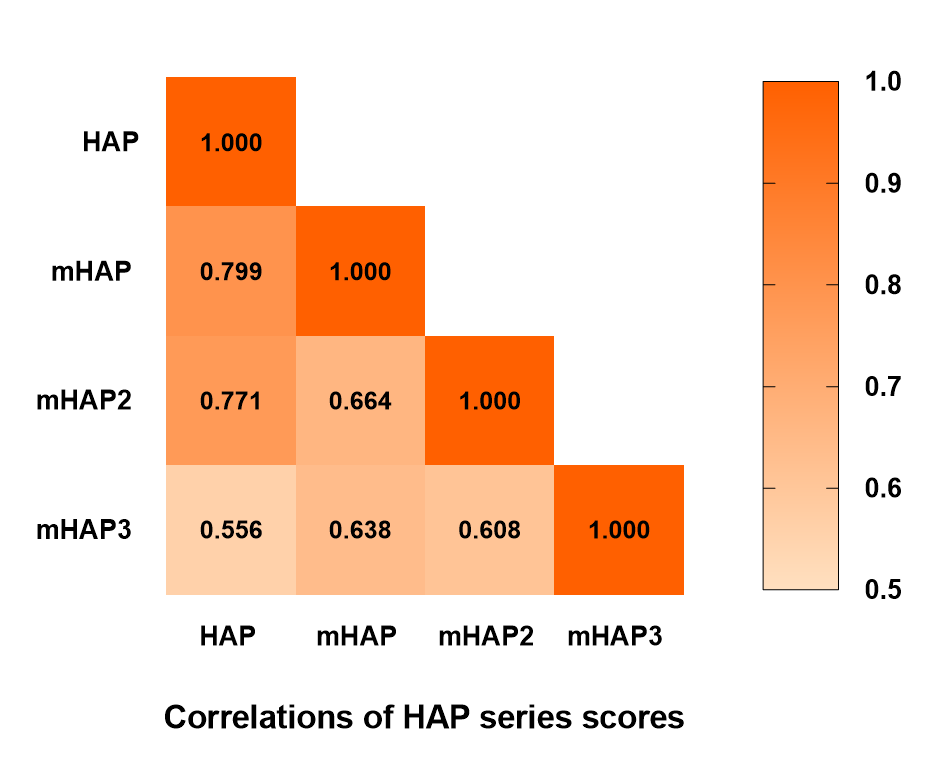

Supplement: Supplementary file 1 [file DataSheet_1.docx]
